# Moisture Absorption Characteristics of Nanoparticle-Doped Silicone Rubber and Its Influence Mechanism on Electrical Properties

**DOI:** 10.3390/polym13091474

**Published:** 2021-05-02

**Authors:** Xiaoqian Zhu, Yunxiao Zhang, Yuanxiang Zhou, Xin Huang

**Affiliations:** 1 State Key Laboratory of Power System and Generation Equipment & Department of Electrical Engineering, Tsinghua University, Beijing 100084, China; zhuxq19@mails.tsinghua.edu.cn (X.Z.); zhangyx09@foxmail.com (Y.Z.); x-huang17@mails.tsinghua.edu.cn (X.H.); 2 The Wind Solar Storage Division of State Key Lab of Control and Simulation of Power System and Generation Equipment School of Electrical Engineering, Xinjiang University, Urumqi 830047, China

**Keywords:** silicone rubber, moisture absorption, space charge, TGA, interface

## Abstract

To further explore the long-term stability of nano-dielectrics, experiments were carried out to investigate the moisture absorption characteristics and electrical properties of silicone rubber (SiR) doped with different inorganic nanoparticles. Thermogravimetric analysis (TGA) is utilized to research the moisture absorption characteristics including mass fraction and binding forms. The trap depth and electron orbitals are calculated by density functional theory to explain the influence mechanism of water molecules on SiR. It is found that the addition of nanoparticles will increase the moisture content of SiR. Additionally, the nano-TiO_2_-doped SiR absorbs more water and binds with water relatively more loosely than nano-Al_2_O_3_. There is a degradation of space charge inhibition capability and breakdown strength after moisture absorption, which might be explained by shallow traps brought by water molecules and the narrowed forbidden bandwidth of SiR.

## 1. Introduction

With the development of high-voltage direct-current (HVDC) transmission technology and the continuous improvement of voltage levels, the performance of silicone rubber (SiR) for cable accessories is far from keeping up with the demand. Furthermore, the breakdown rate of cable accessories is much higher than that of the cable body [1,2,3,4,5]. Therefore, it is necessary to improve the dielectric properties of SiR. With the boom of nano-dielectric research, abundant studies have shown that adding a few nanoparticles to the base material can significantly affect the dielectric properties [6,7,8,9,10]. Researchers have also tried to improve the electrical properties of SiR by adding nanoparticles. Shang et al. made the electrical conductivity of SiR consistent with that of the cross-linked polyethylene (XLPE) through nano-modification, thereby improving the electrical field distribution near the stress cone [11]. Chen et al. found that nano-TiO_2_ doped SiR had a significant effect on inhibiting the accumulation of space charge [12]. Furthermore, Zhou et al. found that Al_2_O_3_/SiR and MgO/SiR both showed strong thermal aging resistance [13].

However, nano-dielectrics have not yet been widely used in practical engineering, which is closely related to the instability of nano-dielectrics [14]. Therefore, while focusing on the short-term performance of nano-dielectrics, more attention should be paid to the long-term service performance. Cable trenches are usually in a state of high temperature and humidity in summer, and cables in coastal areas have been in a state of high humidity for a long time [15,16]. It is known that the surface of the nanoparticles, which exerts tremendous interface advantages in dielectric properties, has a much stronger water absorption capability than polymer. Therefore, it is necessary to consider the moisture absorption characteristics of nanocomposites and its influence on dielectric properties, which will further verify the possibility of nano-dielectric engineering applications.

Researchers are increasingly paying attention to the moisture absorption characteristics of nano-dielectrics. Fabiani et al. found that when the vinyl acetate nanocomposite was damp, the space charge accumulation increased and the breakdown strength decreased [17]. Hui et al. found that the damp SiO_2_/XLPE nanocomposite had worse breakdown strength and more losses [15]. Yang et al. found that under a high electric field, moisture absorption decreased the dielectric properties of the MgO/LDPE nanocomposite, while under a low electric field, the nanocomposites maintained the ability to inhibit the accumulation of space charge [18]. Chi et al. found that the performance of polypropylene-based (PP) nano-dielectrics degraded obviously in a humid environment [19]. To the best of our knowledge, there are few reports on the moisture absorption characteristics of nano-doped SiR. Moreover, the influence mechanism of the moisture on the charge transportation is still unclear. 

Therefore, this paper studies the moisture absorption characteristics and electrical properties after moisture absorption of different types of nano-doped SiR. Moreover, the reason for the degradation of electrical properties and the influence mechanism of water molecules were analyzed. It is expected to provide a reference for the engineering application of nano-composite silicone rubber.

## 2. Materials and Methods

### 2.1. Sample Preparation 

Nano Alumina (Al_2_O_3_) (particle size: 30 nm, purity: 99.9%) is from Beijing Deke Daojin Science And Technology Co., Ltd, Beijing, China. Nano Titanium oxide (TiO_2_) (particle size: 30 nm, purity: 99.9%) is from Shanghai Keyan Industrial Co., Ltd, Shanghai, China. Liquid SiR R6040 is from ZhongLanChenguang Chemical Research Institute Co., Ltd, Chengdu, China.

The preferred mass fraction of 4% of nanoparticles was added to the liquid SiR. Then, the nano-doped SiR was stirred evenly with a high-speed stirrer and vacuumed by a vacuum drying oven. After that, the nano-doped SiR was compressively molded in the condition of 165 °C, 5 MPa provided by flat curing press for 10 min. The thickness of the samples for dielectric properties experiments was about 500 μm. To improve the measurement accuracy by enlarging the mass increment, the thickness of samples for moisture absorption experiments was about 3 mm.

### 2.2. Moisture Absorption Experiment

First, three sets of samples of SiR, nano alumina doped SiR (Al_2_O_3_/SiR), and nano titanium oxide doped SiR (TiO_2_/SiR) were vacuum dried at 80 °C for 120 h, and the samples’ weight was recorded as the original weight. Then, the moisture absorption experiment was carried out in a constant temperature and humidity box where the temperature was 20 °C and humidity was 100% RH. There were 3 samples in each group, and they were taken out and measured 3 times repeatedly every 48 h. According to the standard ISO 12570–2000, if the error of three consecutive measurements was less than 0.1%, the samples were considered as saturated. The moisture absorption curves were obtained by taking the average of the samples from each group.

### 2.3. Characterization

The dry and damp nanoparticles were characterized by TGA and Fourier transform infrared (FTIR). TGA was performed under nitrogen conditions using TGA550 (TA Instrument Co., Ltd, New Castle, US) at 30–800 °C with a heating rate of 20 °C min^−1^. The FTIR measurement range was 4000–600 cm^−1^, and its resolution was 2 cm^−1^.

The JSM-6335 (JEOL Ltd., Tokyo, Japan) field emission scanning electron microscope (FESEM) was used to observe the morphology of the fracture surface of the nanocomposites to determine the dispersion of nanoparticles in the composite.

### 2.4. DC Breakdown Strength

The DC breakdown strength of samples was determined according to IEC 60243. The test was carried out in insulating oil using spherical electrodes (d = 20 mm) and plate electrodes (d = 25 mm) made of stainless steel. The measurement was performed at 30 °C under oven control. Before testing, the oven was preheated for at least 2 h to ensure uniform temperature distribution. The negative DC voltage was applied to the samples, and its increasing rate was 1 kV/s. Each group was measured 15 times to confirm the validity of the data, which were finally expressed in the Weibull distribution.

### 2.5. Space Charge Distribution 

The space charge distribution was measured based on the pulse electroacoustic (PEA) method. The detailed information on PEA measurement can be found in [20]. The polarization process was carried out at −20 kV/mm for 30 min at room temperature. Moreover, the time of depolarization was 10 min.

### 2.6. Density Functional Theory and Trap Calculation 

Density functional theory (DFT) has been widely used in the calculation of charge density distribution and electron affinity [21,22]. The depth of the traps brought by water molecules is calculated by density functional theory, by which a correlation can be established between the space charge trap depth of polymer molecules and the electron affinity. The electron affinity can be regarded as the energy change of adding electrons or extracting holes to the molecule.

The depth of the traps brought by water molecules is calculated by density functional theory, by which a correlation can be established between the space charge trap depth of polymer molecules and the electron affinity. The electron affinity can be regarded as the energy change of adding electrons or extracting holes to the molecule [23,24]. The electron affinity of the molecule can be calculated by Equation (1):

(1) $$EA=E\left(Re\right)-E^{-}\left(Re^{-}\right)$$

In Equation, $E\left(Re\right)$ represents the total energy of neutral molecules in a stable configuration. $E^{-}\left(Re^{-}\right)$ represents the total energy of anionic molecules with a negative charge in a stable configuration.

In solid dielectrics, the electron affinity is the energy required to bring electrons from the vacuum into the conduction band. Moreover, the electron affinity of the defect is the energy required to bring electrons from the vacuum level to the defect level. Therefore, the trap depth $E_{trap}$ refers to the difference in electron affinity between a defective system and a non-defective system.

(2) $$E_{trap}=EA_{defect}-EA_{reference}$$

## 3. Results

### 3.1. Characterization and Moisture Absorption

**Figure 1:**
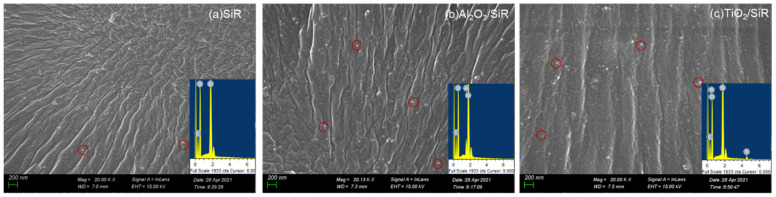
SEM images of the fractured faces of the samples: (**a**) SiR, (**b**) Al_2_O_3_/SiR, (**c**) TiO_2_/SiR.

The SEM images of different nanocomposite silicone rubbers are shown in Figure 1. The white points in the SiR matrix represent the nanoparticles, which are partially circled by red rings. The energy dispersive spectrum (EDS) was confirmed the addition of corresponding nanoparticles. The SEM images reflect that the nanoparticles are well dispersed in the SiR, and there is no obvious agglomeration phenomenon. 

The water absorption *H%* is expressed as the mass percentage (wt%) relative to the dry sample, as shown in Equation (3):

(3) $$H\%=\frac{m-m_{0}}{m_{0}}\times 100\%$$

**Figure 2:**
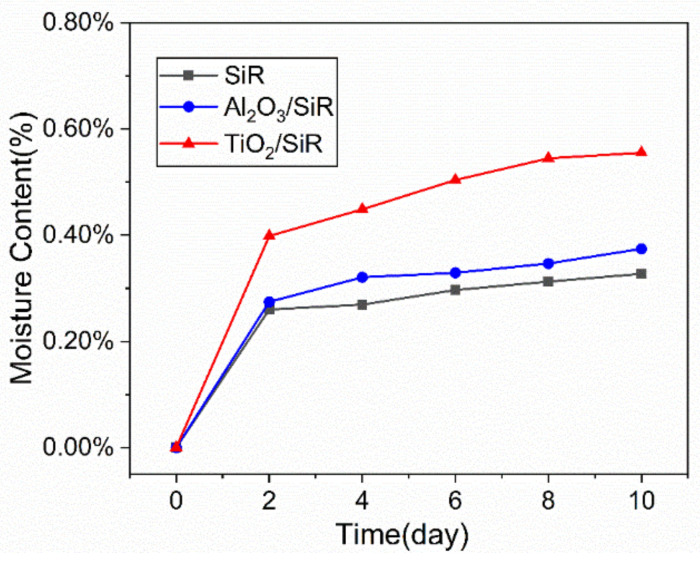
Moisture content of different composites.

In the Equation, *m* is the mass of the damp samples, and *m*_0_ is the mass of the original dry samples. The moisture absorption characteristics of different composites is shown in Figure 2. The moisture absorption capability of SiR increases after adding nanoparticles. The saturated moisture content of SiR is about 0.33%, while the moisture content of Al_2_O_3_/SiR and TiO_2_/SiR is 0.37% and 0.56%, respectively. The TiO_2_/SiR has the strongest water absorption capability, while Al_2_O_3_/SiR exhibits weaker moisture absorption capability. It can be inferred that the moisture absorption capability is closely related to the properties of the nanoparticles.

**Figure 3:**
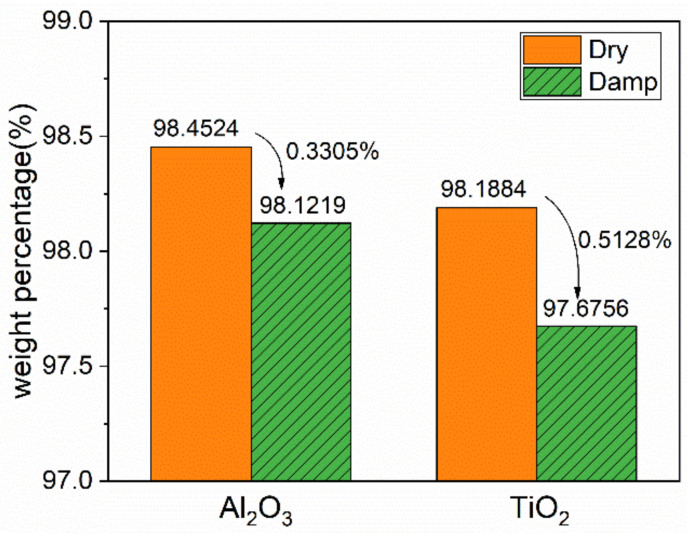
Remaining weight percentage of nanoparticles after TGA experiment.

To further verify that the moisture absorption characteristics of nano-doped SiR are indeed caused by nanoparticles, TGA of dry and damp nanoparticles alone is done. The remaining mass of dry and damp nanoparticles is shown in Figure 3. It can be seen that the weight loss of Al_2_O_3_ and TiO_2_ nanoparticles caused by moisture absorption is 0.3305% and 0.5128%, respectively, which can reflect the moisture absorption capability of nanoparticles. By comparison, the moisture content of nanoparticles is slightly lower than that of nano-doped SiR, since the original liquid SiR will absorb water in high-humidity environments. However, their trend of moisture absorption capacity is the same, which verifies that the characteristics of nanoparticles influence the moisture absorption characteristics of the nanocomposite.

**Figure 4:**
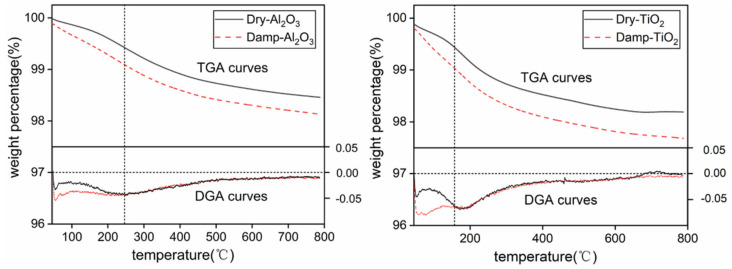
TGA and DGA curves of nanoparticles.

TGA and differential thermogravimetric analysis (DGA) curves of dry and damp Al_2_O_3_ and TiO_2_ nanoparticles are shown in Figure 4. The temperature point where the DGA curves of dry and damp nanoparticles intersect and remain coincident is defined as the highest binding temperature in this paper. Before the highest binding temperature is reached, the thermal weight loss rate of damp nanoparticles is faster than that of dry nanoparticles. The overlapping DGA curves indicate that the thermal weight loss difference caused by moisture absorption is mainly concentrated before this temperature. 

There are three components of water in nanoparticles after moisture absorption, namely free water molecule, weakly bonded water, and strongly bonded water [18]. It is generally considered that the more closely the water molecules and nanoparticles combine, the harder it is for them to separate. The highest binding temperature of Al_2_O_3_ and TiO_2_ is about 240 °C and 160 °C, respectively. Therefore, it is believed that the binding form of these two kinds of nanoparticles and water may be different, which means Al_2_O_3_ nanoparticles and water molecules may be more tightly combined and are more likely to form bonded water.

**Figure 5:**
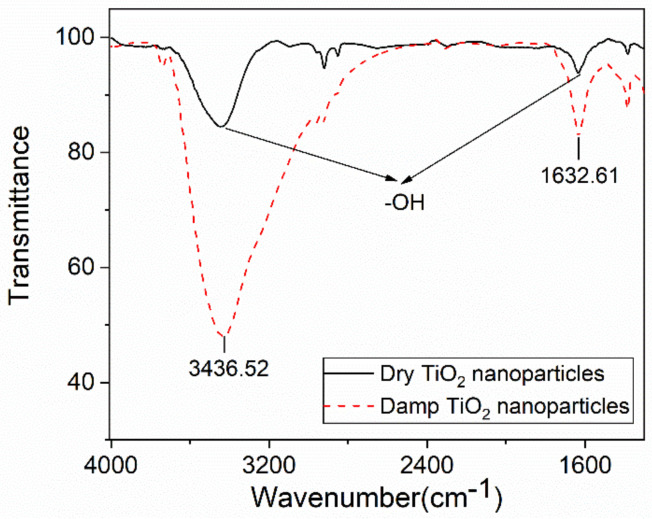
FTIR spectra of TiO_2_ nanoparticles.

Figure 4 also shows that the main weight loss is not caused by moisture absorption as the weight loss of dry nanoparticles is more than 1%. FTIR is used to further characterize the surface of the nanoparticles. As shown in Figure 5, the FTIR result of TiO_2_ is taken as a representative. The wavenumber 3400 cm^−1^ and 1636 cm^−1^ represent the bending vibration and stretching vibration peaks of the hydroxyl group, respectively, corresponding to the hydroxyl group on the surface of the TiO_2_ nanoparticles [25]. The infrared spectrum of TiO_2_ shows that the damp nanoparticles have introduced a large number of hydroxyl groups. It is worth noting that the dry nanoparticles also contain hydroxyl groups and some other groups, which may contain some by-products, which may be the reason for the weight decline of dry nanoparticles.

### 3.2. Space Charge Characteristics

**Figure 6:**
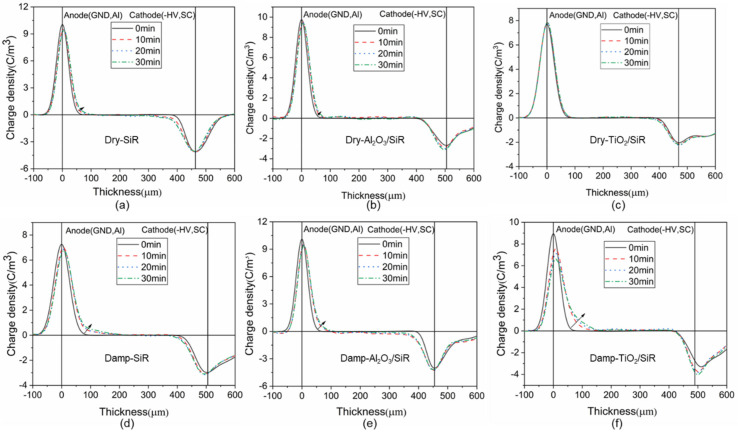
Space charge distribution of SiR (**a**) Dry SiR, (**b**) Dry Al_2_O_3_/SiR, (**c**) Dry TiO_2_/SiR, (**d**) Damp SiR, (**e**) Damp Al_2_O_3_/SiR, (**f**) Damp TiO_2_/SiR.

The space charge characteristics of dry and damp nano-doped SiR were obtained by the PEA method. As shown in Figure 6, the first row represents the space charge accumulation of dry nanocomposite SiR, while the second row represents damp SiR. It can be seen from Figure 6a that there are some homo-charges accumulated in the SiR. The nano-Al_2_O_3_-doped SiR has no obvious effect on inhibiting the injection of space charge, while the nano-TiO_2_ has a significant inhibition effect, which is consistent with previous work [12,26]. 

Comparing the space charge of dry and damp nanocomposite SiR, the space charge inhibition capability of nano-doped SiR dropped dramatically after moisture absorption. The space charge injection of the damp nano-TiO_2_ doped SiR is the most obvious, which is even worse than that of damp SiR, losing the advantages brought by nanoparticles under dry conditions.

Takada [27] used a potential well model composed of dipoles induced by high-dielectric nanoparticles to explain the space charge inhibition effect. The potential well induced by high-dielectric nanoparticles is about 1.5 ~ 5 eV, which will trap and hinder the movement of space charge carriers, thereby inhibiting the accumulation of space charges in the nanocomposite dielectric. Therefore, this paper speculates that nano-TiO_2_ with a high dielectric constant introduces deep traps in the nanocomposites, in which there is almost no space charge accumulation. Compared with SiR, nano-Al_2_O_3_ has no significant space charge inhibition ability. This may be because that the depth of the trap introduced by the nano-Al_2_O_3_ is not deep enough to hinder the movement of space charge carriers as the Al_2_O_3_ nanoparticles have a much lower dielectric constant.

**Figure 7:**
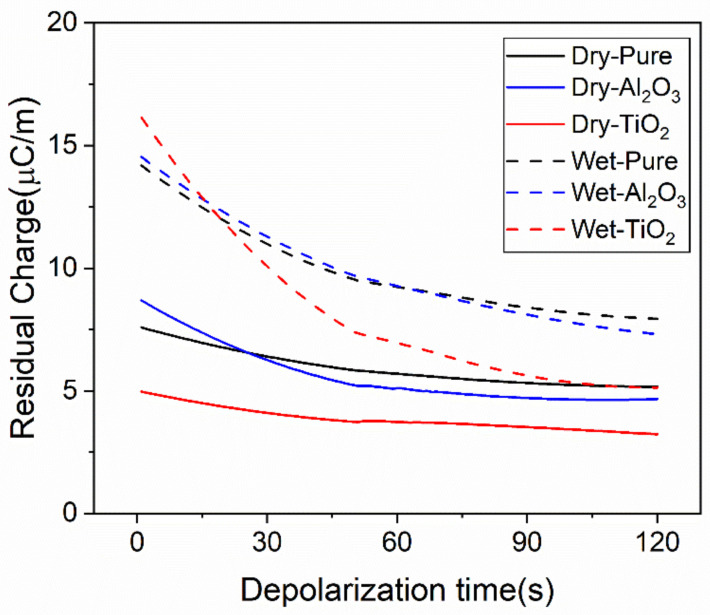
Residual charge of dry and damp SiR.

The space charge distribution during depolarization is measured to further analyze the potential wells of nano-doped SiR. Since the induced charge in the polarization process will affect the observation of the charge accumulated in the dielectrics, the space charge distribution in the depolarization process can more truly reflect the amount and distribution of space charge. In addition, it can reflect the trap depth of the nano-doped SiR [28,29]. By integrating the space charge density distribution during the depolarization process, the change in residual charge with time is shown in Figure 7.

It is seen that the quantity of residual charge of the damp SiR is generally larger than that of dry SiR. The damp TiO_2_/SiR has the largest amount of residual charge and the fastest charge dissipation rate, indicating that the water molecules have introduced numerous shallow traps as they make it easier for space charge to accumulate and dissipate. In comparison, the damp Al_2_O_3_/SiR has less residual charge, which might be attributed to the relatively weaker moisture absorption capability. Furthermore, the quantity of stable residual charge of Al_2_O_3_/SiR after depolarization for 10 min is more than that of damp TiO_2_/SiR and the charge dissipation rate is slower, indicating that the Al_2_O_3_/SiR introduces more relatively deep traps. However, the depth of these traps may not be sufficient to inhibit space charge injection.

The TGA results above show that these two kinds of nanoparticles bind differently with water molecules. The quantity and dissipation rate of residual charge reflect the different trap levels in nano-doped SiR. It is inferred that the depth of the traps is related to the binding form of nanoparticles and water molecules. That is, the relatively deeper traps in damp Al_2_O_3_/SiR are brought by the tightly bound water molecules.

### 3.3. DC Breakdown Strength

**Figure 8:**
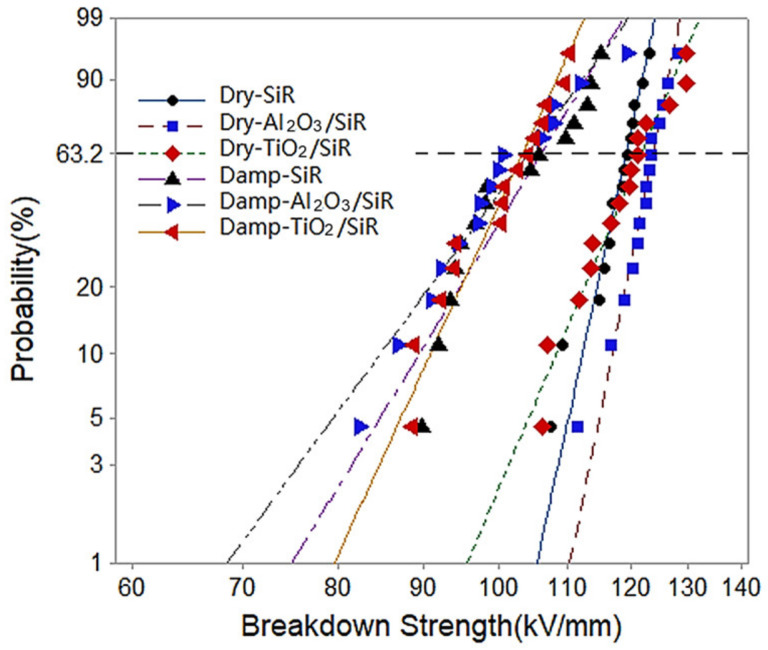
Weibull distribution of the DC breakdown strength of SiR composite.

Experiments are also carried out to investigate the influence of moisture absorption on the DC breakdown strength of SiR at room temperature. As Figure 8 shows, the breakdown strength of dry nano-doped SiR remains unchanged or slightly increases. However, the breakdown strength degrades after moisture absorption. Moreover, the breakdown strength drop rate of Al_2_O_3_/SiR and TiO_2_/SiR is 15.93% and 15.20%, respectively, which is larger than that of SiR. The specific values are shown in Table 1.

### 3.4. Trap Depth and Electronic Orbital Distribution

To further analyze the mechanism of moisture absorption on the electrical properties, density functional theory is used to calculate the trap depth and orbital of SiR. To simplify the model, the trap level affected by water molecules alone is calculated. The comprehensive effect of nanoparticles and water molecules will be discussed later. The calculated result is shown in Table 2. The trap depth brought by water molecules is 0.39 eV, which is regarded as a shallow trap. The theoretical calculation is consistent with the trend reflected by the space charge results.

**Figure 9:**
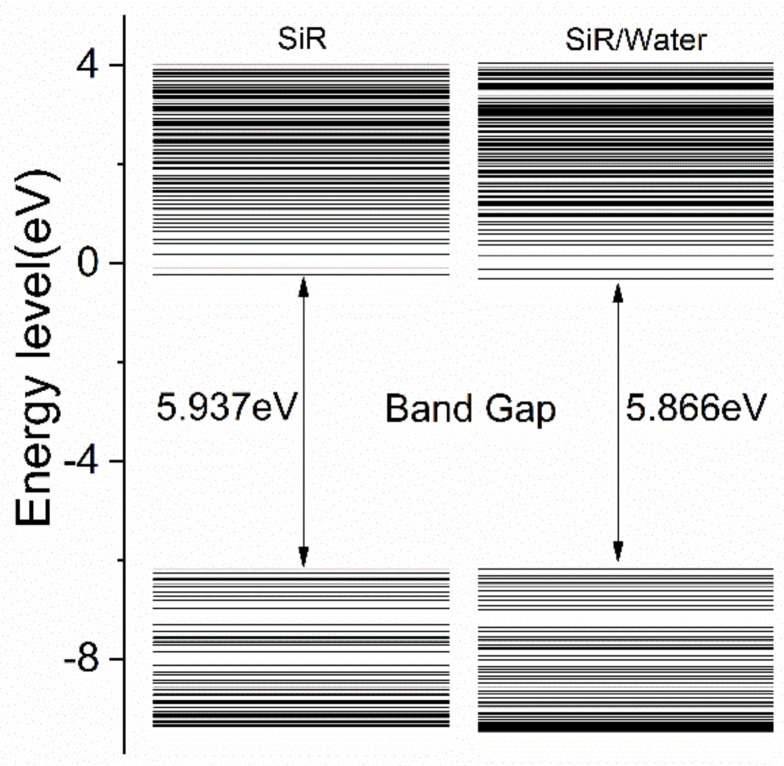
Electron Orbital distribution of SiR with moisture.

Density functional theory was used to calculate the electronic orbital distribution of SiR with water molecules. It can be seen from Figure 9 that the highest occupied molecular orbital energy level of the system is reduced, thereby narrowing the forbidden band gap of the SiR, resulting in a decrease in insulation performance.

## 4. Discussion

In the moisture absorption experiment, the moisture content of TiO_2_/SiR was more than that of nano-Al_2_O_3_/SiR. However, there was almost no difference in the breakdown strength between these two after moisture absorption. Similar to the results of space charge, it is inferred that breakdown strength after moisture absorption is not only related to moisture content but also related to the way water molecules exist in the polymer. From the TGA results above, it can be seen that Al_2_O_3_ nanoparticles are more tightly bound with water molecules than TiO_2_ nanoparticles. It may be guessed that the binding ways of water molecules and nanoparticles may have an influence on the interface, which is considered to be the main reason for the improvement of the nanocomposites [8,30,31].

**Figure 10:**
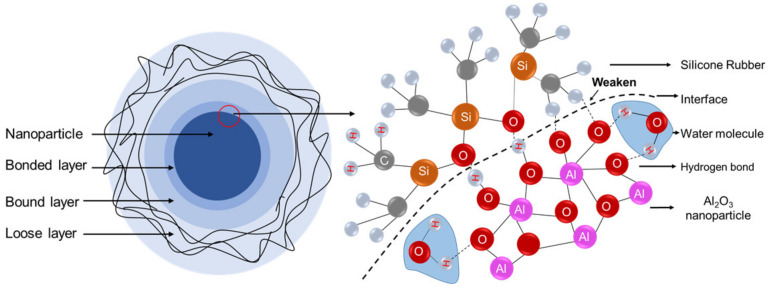
Interface of nanoparticles and SiR.

The schematic of the interface between nanoparticles and polymer is drawn according to the multi-core model proposed by Tanaka [32]. As shown in Figure 10, there is the bonded layer, the bound layer, and the loose layer from the surface of the nanoparticle to the outside. Taking Al_2_O_3_/SiR as an example, the right part of the figure is the partial magnification of the bonded layer, where the atoms on the surface of Al_2_O_3_ form chemical bonds with SiR [33]. As the nanoparticles have surface hydrophilicity, the water molecules may penetrate into the bonded layer and form a new chemical bond, which may weaken the original bonds and affect the interface area [34]. It is believed that the bonding between nanoparticles and base material is thermodynamically unstable in the presence of water molecules, and there is a driving force for the displacement of adhesive on the interface between particles and matrix by water [35].

Based on these discussions, it is believed that the water molecules tightly bound with nanoparticles affect the insulation performance by undermining the interface. Therefore, under the comprehensive effect of water content and binding form, the final performance shows almost no difference in the breakdown performance of SiR added with TiO_2_ and Al_2_O_3_.

## 5. Conclusions

In this study, the moisture absorption characteristic of nanoparticle-doped SiR and its influence mechanism on electrical properties were investigated. The main findings are as follows.

1. The addition of nanoparticles increases the water absorption of SiR. The moisture absorption capability and the binding forms with water molecules of nanoparticles are different. TiO_2_ nanoparticles have stronger moisture absorption capability, but they bind relatively loosely with water molecules. Al_2_O_3_ nanoparticles have lower moisture absorption capability than TiO_2_, but they are more closely bound to water molecules.

2. TiO_2_/SiR has significant space charge inhibition ability under dry conditions. However, it completely loses its advantage after moisture absorption. The water molecules bring shallow traps in the SiR, and the forbidden band gap is narrowed, which may explain the degradation of space charge inhibition ability and breakdown strength of nano-doped SiR after moisture absorption.

3. The different binding forms account for the differences in electrical properties of different nano-doped SiR after moisture absorption. The invasion of tightly binding water molecules may weaken the original bond and undermine the interface of SiR and nanoparticles.

## Figures and Tables

**Table 1:** Comparison of DC breakdown strength of dry and wet samples.

Sets	Dry Sample (kV/mm)	Damp Samples (kV/mm)	Drop Rate
SiR	119.2	105.9	11.16%
Al_2_O_3_/SiR	123.7	104.0	15.93%
TiO_2_/SiR	121.7	103.2	15.20%

**Table 2:** Electron affinity and trap depth of H_2_O/SiR.

Sets	$E\left(Re\right)/\mathrm{eV}$	$E^{-}\left(Re^{-}\right)/\mathrm{eV}$	EA/eV	$E_{trap}/\mathrm{eV}$
Reference(SiR)	−440.76	−440.26	−0.50	Null
Defect(H_2_O/SiR)	−494.36	−494.25	−0.11	0.39

## Data Availability

The data presented in this study are available on request from the corresponding author.
